# Time trends in municipal distribution patterns of cancer mortality in Spain

**DOI:** 10.1186/1471-2407-14-535

**Published:** 2014-07-24

**Authors:** Gonzalo López-Abente, Nuria Aragonés, Beatriz Pérez-Gómez, Marina Pollán, Javier García-Pérez, Rebeca Ramis, Pablo Fernández-Navarro

**Affiliations:** Environmental and Cancer Epidemiology Unit, National Centre for Epidemiology, Carlos III Institute of Health, Avda, Monforte de Lemos 5, 28029 Madrid, Spain; Consortium for Biomedical Research in Epidemiology and Public Health (CIBER en Epidemiología y Salud Pública - CIBERESP), Madrid, Spain

**Keywords:** Disease mapping, Cancer mortality, Epidemiology, Spatial epidemiology

## Abstract

**Background:**

New disease mapping techniques widely used in small-area studies enable disease distribution patterns to be identified and have become extremely popular in the field of public health. This paper reports on trends in the geographical mortality patterns of the most frequent cancers in Spain, over a period of 20 years.

**Methods:**

We studied the municipal spatial pattern of stomach, colorectal, lung, breast, prostate and urinary bladder cancer mortality in Spain across four quinquennia, spanning the period 1989-2008. Case data were broken down by town (8073 municipalities), period and sex. Expected cases for each town were calculated using reference rates for each five-year period. For map plotting purposes, smoothed municipal relative risks were calculated using the conditional autoregressive model proposed by Besag, York and Mollié, with independent data for each quinquennium. We evaluated the presence of spatial patterns in maps on the basis of models, calculating the variance in relative risk corresponding to the structured spatial component and the unstructured component, as well as the proportion of variance explained by the structured spatial component.

**Results:**

The mortality patterns observed for stomach, colorectal and lung cancer were maintained over the 20 years covered by the study. Prostate cancer and the tumours studied in women showed no defined spatial pattern, with the single exception of stomach cancer. The trend in spatial fractional variance indicated the possibility of a change in the spatial pattern in breast, bladder and colorectal cancer in women during the last five-year period. The paper goes on to discuss ways in which spatio-temporal data are depicted in the case of cancer, and review the risk factors that may possibly influence the respective tumours’ spatial patterns.

**Conclusion:**

In men, the marked geographical patterns of stomach, colorectal, lung and bladder cancer remained stable over time. Breast, colorectal and bladder cancer in women show signs of the possible appearance of a spatial pattern in Spain and should therefore be monitored.

**Electronic supplementary material:**

The online version of this article (doi:10.1186/1471-2407-14-535) contains supplementary material, which is available to authorized users.

## Background

New disease mapping techniques widely used in small-area studies enable disease distribution patterns to be identified and have become extremely popular in the field of public health [1, 2]. Cancer and other disease mortality atlases [3–5] have shown that many risk factors of a territorial (and thus of an environmental) nature, influence geographical patterns. New methods of analysis make it possible to smooth selected disease indicators and so reveal their geographical structure [6]. Estimators obtained in less populated areas share information with neighbouring areas, assuming that they are exposed to common environmental factors. Yet, disease incidence and mortality are dynamic processes and therefore variable in space and time. As a result, different spatio-temporal disease mapping techniques have recently been proposed without any broad consensus as to how to describe spatio-temporal disease trends [7–11].

The aim of this study was to report on trends in geographical mortality patterns of the most frequent cancers in Spain across four quinquennia, and in so doing, update the information published in previous studies [12–14].

## Methods

As our case source, we used individual death entries for the period 1989-2008 corresponding to stomach cancer (International Classification of Diseases, Ninth Revision (ICD-9) code 151, ICD-10 C16), colorectal cancer (ICD- 9 codes 153-154, 159.0, ICD-10 code C18-C21), lung cancer (ICD- 9 code 162, ICD-10 code C33-C34), breast cancer in women (ICD- 9 code 174, ICD-10 code C50), prostate (ICD- 9 code 185, ICD-10 code C61) and bladder cancer (ICD- 9 code 188, ICD-10 code C67). These data were furnished by the National Statistics Institute (*Instituto Nacional de Estadística* - *INE*). The observed case data were broken down by town (8073 municipalities) and sex. Municipal populations, with a breakdown by age group (18 groups) and sex, were obtained from the 1991 and 2001 censuses and the 1996 and 2006 municipal rolls, official information provided by INE. These years corresponded to the midpoints of the four quinquennia comprising the study period (1989-1993, 1994-1998, 1999-2003 and 2004-2008). The person-years for each five-year period were obtained by multiplying these populations by 5.

To calculate expected cases, the overall Spanish mortality rates for the above four 5-year periods were multiplied by each town’s person-years, by age group and quinquennium. Standardised mortality ratios (SMRs) were calculated as the ratio of observed to expected deaths.

For map plotting purposes, smoothed municipal relative risks (RRs) were calculated using the conditional autoregressive model proposed by Besag, York and Mollié (BYM) [6]. This model is based on fitting a Poisson spatial model with observed cases as the dependent variable, expected cases as offset, and two types of random effect terms which take the following into account: a) municipal contiguity (spatial term υ_i_); and b) municipal heterogeneity (ν_i_). α is the intercept quantifying the average mortality rate in all the towns. In this model the linear predictor is:


The smoothed RRs for plotting purposes are (ζ _i_ = exp(υ_i_ + ν_i_)). A separate model was fitted for each period.

Lastly, in order to have an indicator of the presence of a spatial pattern in the mortality plotted for the respective cancers, we estimated the variance in relative risk corresponding to the structured spatial component and the unstructured component, as well as the proportion of variance explained by the structured spatial component [10].

Integrated nested Laplace approximations (INLAs) were used as a tool for Bayesian inference. For this purpose, we used R-INLA [15] with the option of simplified Laplace estimation of the parameters, a package available in the R environment [16]. A total of 8073 towns were included, and the spatial data on municipal contiguities was obtained by processing the official *INE* maps.

Examples of R scripts describing the models used to obtain the relative risk smoothed maps and the calculation of the components of the variance of spatial terms have been published elsewhere [10].

## Results

**Figure 1:**
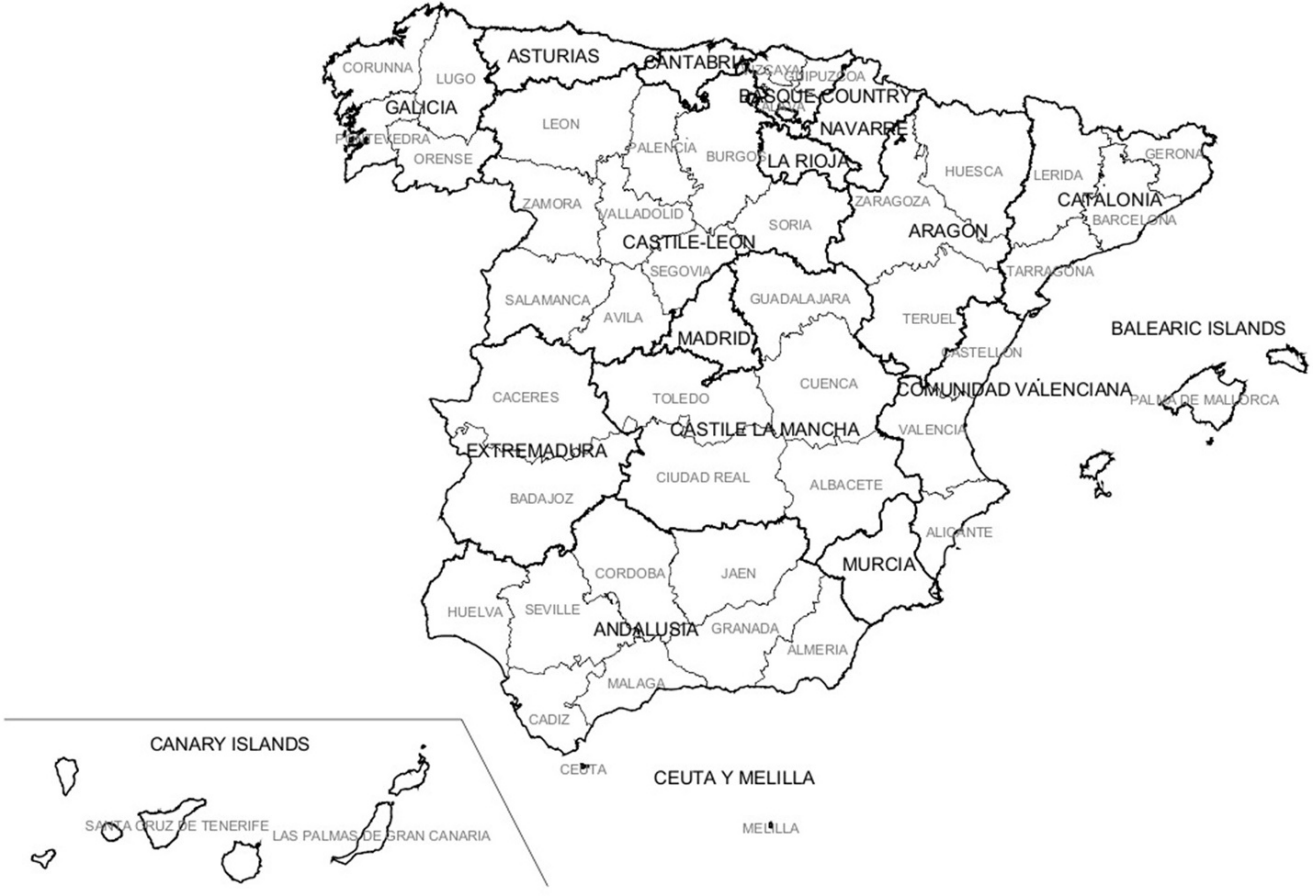
**Geographical situation of Spain’s provinces and Autonomous Regions (** ***Comunidades Autónomas*** **).**

Table 1 shows the number of deaths for the tumours studied, by sex and five-year period, and the trend in age-adjusted rates (European standard population) [17]. Data on the number of deaths are of interest for assessing the figure used to estimate spatial patterns, and the adjusted rates are of interest for assessing the general mortality trend for each tumour. Most of the cancers studied registered a decline in mortality in the last five-year period, except for: colorectal cancer in men (which rose 1.2% in 4^th^ versus the 3^rd^ quinquennium); and lung and bladder cancer in women (which rose 2.6% and 0.6% respectively in 4^th^ versus the 3^rd^ quinquennium). Figure 1 shows the situation of the different provinces in Spain.

**Table 1:** **Deaths & age-adjusted rates (x 100,000) by sex, period and cancer site in Spain**

Cancer site			1989-1993	1994-1998	1999-2003	2004-2008	Total 1989-2008
Stomach cancer	Men	Deaths	20176	19596	18367	17611	75,750
		Rate	21.16	18.22	15.14	12.83	
	Women	Deaths	13681	12616	11606	10908	48,811
		Rate	9.86	8.04	6.59	5.54	
Colorectal cancer	Men	Deaths	23196	28958	33542	38269	123,965
		Rate	24.24	26.68	27.03	27.36	
	Women	Deaths	21378	24976	26036	27950	100,340
		Rate	15.75	16.15	17.79	14.08	
Lung cancer	Men	Deaths	65853	74395	79704	84398	304,350
		Rate	68.95	70.04	67.52	64.22	
	Women	Deaths	6827	8076	9967	13335	38,205
		Rate	5.35	5.84	6.85	8.64	
Breast cancer	Women	Deaths	27638	29117	28866	29459	115,080
		Rate	24.53	23.12	20.27	18.38	
Prostate cancer		Deaths	22310	27029	27998	27618	104,955
		Rate	23.32	24.27	21.37	18.43	
Bladder cancer	Men	Deaths	13343	14851	16833	18686	63,713
		Rate	13.81	13.48	13.30	13.07	
	Women	Deaths	2975	3138	3574	4012	13,699
		Rate	2.00	1.77	1.75	1.76

### Stomach cancer

**Figure 2:**
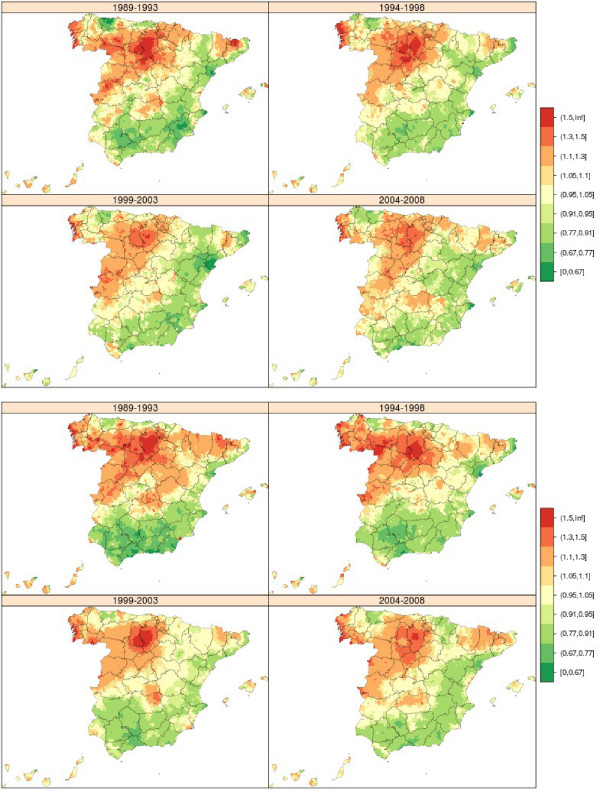
**Municipal distribution of relative risk of stomach cancer mortality in men (above) and women (down) for each five-year period, Independent maps for each quinquennium.** Spain 1989-2008.

During the period 1989-2008, there were 144,561 stomach cancer-related deaths in Spain (75,750 in men and 48,811 in women), accounting for 8% of all deaths due to malignant tumours. Figure 2 shows the maps depicting the municipal distribution of stomach cancer mortality for each quinquennium, using the respective reference rates for men and women. The pattern proved similar for both sexes. These maps clearly show that the geographical pattern changed very little over the course of the 20 years. In general, there was a reduction in the number of towns in the highest RR category. When compared to the average for Spain, areas that maintained an excess risk of dying of gastric cancer continued to be large areas of Castile-Leon and towns along the Atlantic coast of Galicia, with the latter registering higher RRs than those recorded for the provinces of Burgos and Palencia.

### Colorectal cancer

**Figure 3:**
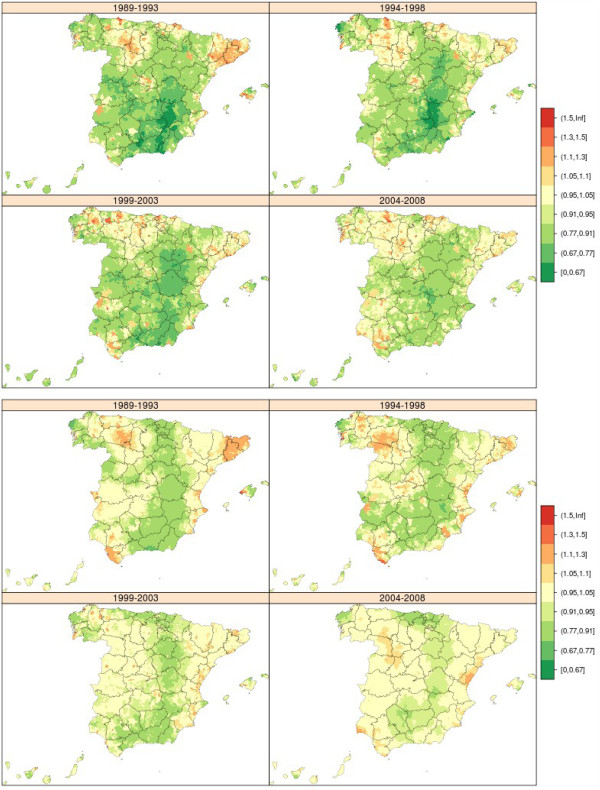
**Municipal distribution of relative risk of colorectal cancer mortality in men (above) and women (down) for each five-year period, Independent maps for each quinquennium.** Spain 1989-2008.

During the period 1989-2008, there were 224,305 deaths due to this cause in Spain (123,965 in men and 100,340 in women), accounting for 14.5% of all deaths due to malignant tumours. Figure 3 shows the maps depicting the municipal distribution of colorectal cancer mortality for each quinquennium, using by way of reference the overall mortality in each period among men and women respectively. The geographical pattern was not very pronounced for this cancer and displayed many similarities between the sexes. The most characteristic feature was that the first five-year period in men and the first two five-year periods in women were marked by excess mortality in towns in Catalonia and in the province of Leon, a pattern that became attenuated in subsequent quinquennia.

### Lung cancer

**Figure 4:**
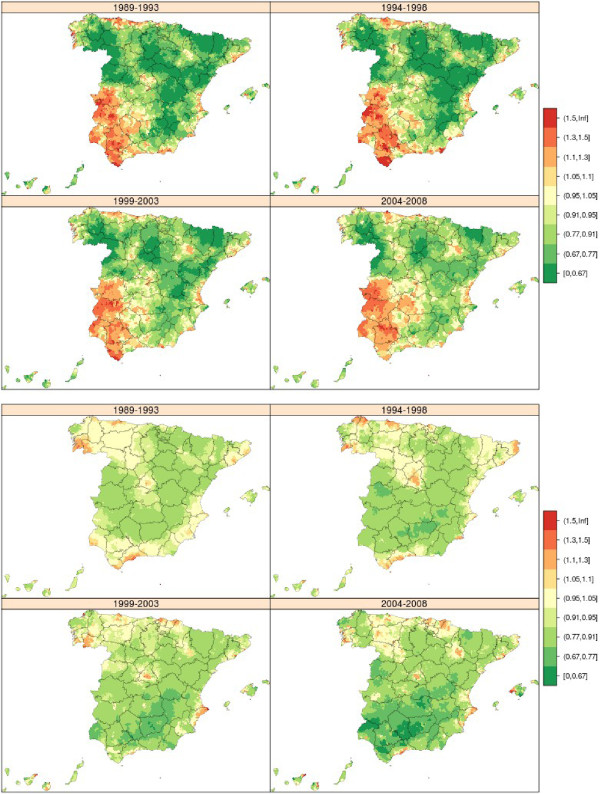
**Municipal distribution of relative risk of lung cancer mortality in men (above) and women (down) for each five-year period, Independent maps for each quinquennium.** Spain 1989-2008.

From 1989 to 2008 there were 342,555 lung cancer-related deaths in both sexes (304,350 in men and 38,205 in women). The municipal distribution of lung cancer mortality in men and women was very different (Figure 4). The geographical pattern in men changed relatively little over the 20 years of study, though the excess observed in the province of Cadiz became attenuated. The areas with highest mortality were the Region of Extremadura, extensive areas of west Andalusia (Huelva, Seville and Cadiz) and towns along the sections of the Cantabrian coast corresponding to Asturias and Cantabria. In women, there was hardly any discernable geographical pattern but some towns in the provinces of Pontevedra and Ourense registered excess mortality across all four quinquennia.

### Breast cancer

**Figure 5:**
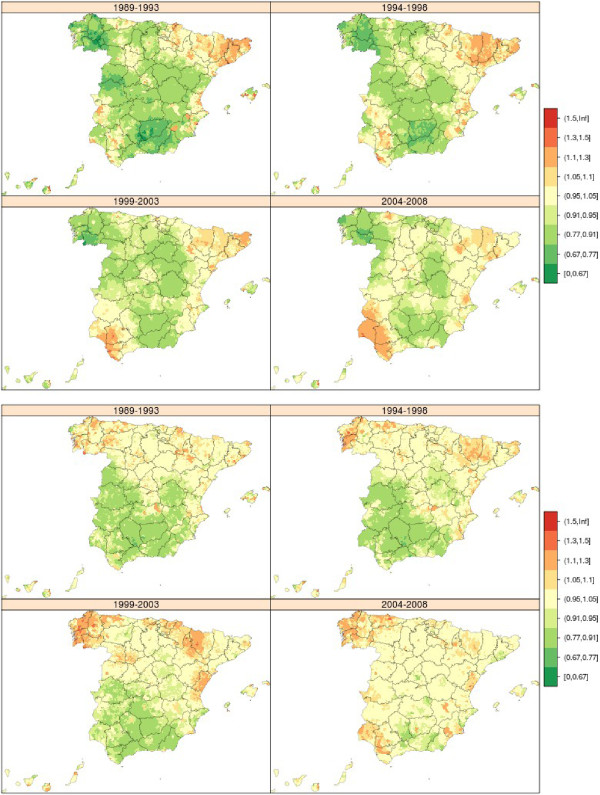
**Municipal distribution of relative risk of breast cancer mortality in women (above) and prostate cancer mortality (down) for each five-year period, Independent maps for each quinquennium.** Spain 1989-2008.

Between 1989 and 2008, there were 115,080 deaths due to breast cancer in women in Spain, which accounted for 18% of female cancer-related deaths and 4% of overall female mortality. There was no unduly pronounced breast cancer mortality pattern (Figure 5 above), indicating that the risk factors were uniformly distributed throughout the territory. During the first two five-year periods, breast cancer in women plotted an already known pattern, marked by towns in Catalonia and the Balearic Isles with higher mortality. Although this excess mortality became somewhat attenuated with time, excess mortality nevertheless emerged across wide swathes of west Andalusia, with towns in Huelva, Seville and Cadiz registering RRs of over 1.10. The lowest mortality was recorded for Galicia, south-west Castile-Leon and east Andalusia.

### Prostate cancer

During the period 1989-2008, there were 104,955 deaths due to prostate cancer in Spain, accounting for 10% of mortality due to all malignant tumours in men. The prostate cancer mortality pattern was not at all pronounced (Figure 5 down), indicating -as in the case of breast cancer among women- territorial uniformity in exposure to possible risk factors. A south-north pattern was in evidence during the first three quinquennia. Galicia was the area having the highest number of towns with excess risk. In Andalusia, which had registered lower than expected mortality in the first three quinquennia, a change seemed to be taking place in the period 2004-2008 in the provinces of Huelva and Cadiz, which began to display RRs of over 1.05 in many towns.

### Bladder cancer

**Figure 6:**
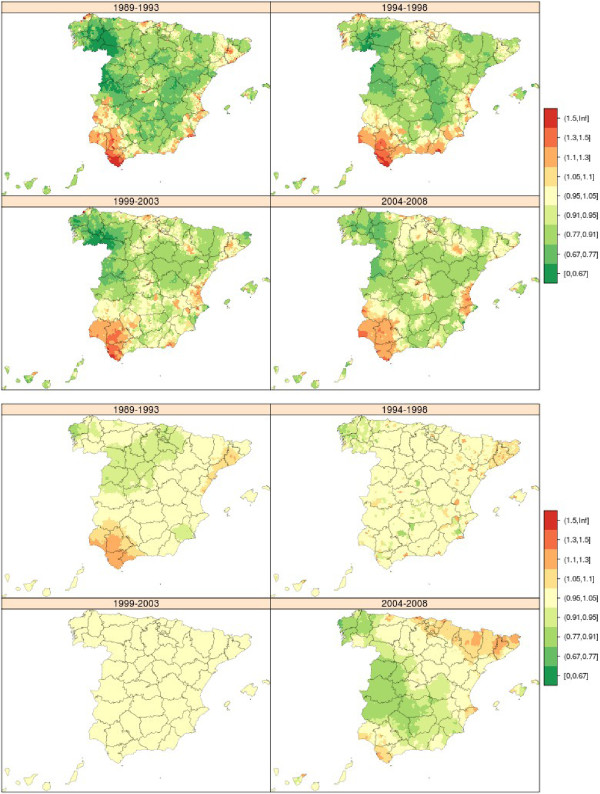
**Municipal distribution of relative risk of bladder cancer mortality in men (above) and women (down) for each five-year period, Independent maps for each quinquennium.** Spain 1989-2008.

From 1989 to 2008, there were 77,412 bladder cancer deaths in Spain, 63,713 in men and 13,699 in women. Although the pattern among men and women was different (Figure 6), with less variability among women, in men the towns with the highest mortality were situated in west Andalusia (Cadiz, Seville, Huelva) and in the province of Barcelona, where excess risk was observed in towns in the Bages district (Suría, Sallent, Balsareny, Manresa and Cardona), which seemed to become attenuated with the passage of time.

Bladder cancer mortality was five times lower among women than among men, with the spatial pattern showing a rather singular trend: in west Andalusia, excess mortality among women was confined to the first five-year period and then disappeared; and, while the pattern displayed by women in towns in Catalonia appeared to level off in the 3^rd^ quinquennium, in the period 2004-2008 there was a resurgence in RRs of over 1.10 in Catalonia and in areas lying in the Pyrenean foothills (Pre-Pyrenees).

### Spatial patterns and changes over time

Table 2 shows the variance in the spatial component, the variance in the component of heterogeneity, and the proportion of the variance in mortality explained by the spatial structure of the models. The magnitude of these values in the spatial component in the model indicates the importance of the spatial pattern. Hence, cancers with the most defined patterns have a proportion of variance explained by the highest spatially structured component. This was the case of lung and bladder cancer in men and stomach cancer in both sexes, with variances in excess of 0.05 in the different periods, which accounted for over 70% of the variance. The presence of a change in the spatial pattern can be evaluated by changes in spatial fractional variance over time. Breast, bladder and colorectal cancer in women display a possible change in the spatial pattern in the last five-year period, a change that will have to be monitored by subsequent studies.

**Table 2:** **Variance in spatial and heterogeneity components, and proportion of the variance in mortality relative risk explained by the spatial structure of the models**

	Men				Women			
Cancer site	1989-93	1994-98	1999-2003	2004-2008	1989-93	1994-98	1999-2003	2004-2008
Stomach								
Spatial	0.083	0.067	0.059	0.053	0.091	0.081	0.063	0.063
Heterog	0.001	0.003	0.009	0.013	0.003	0.007	0.001	0.001
sfv	0.99	0.95	0.86	0.81	0.97	0.92	0.98	0.98
Colorectal								
Spatial	0.040	0.036	0.033	0.017	0.026	0.021	0.011	0.011
Heterog	0.022	0.012	0.020	0.017	0.003	0.009	0.012	0.001
sfv	0.64	0.76	0.62	0.50	0.88	0.70	0.48	0.91
Lung								
Spatial	0.099	0.099	0.076	0.063	0.027	0.038	0.037	0.054
Heterog	0.004	0.002	0.001	0.001	0.003	0.005	0.013	0.015
sfv	0.97	0.98	0.98	0.98	0.90	0.89	0.74	0.79
Breast								
Spatial					0.039	0.028	0.017	0.020
Heterog					0.013	0.008	0.007	0.001
sfv					0.75	0.76	0.70	0.96
Prostate								
Spatial	0.014	0.013	0.014	0.007				
Heterog	0.017	0.012	0.011	0.011				
sfv	0.46	0.52	0.56	0.39				
Bladder								
Spatial	0.067	0.054	0.043	0.045	0.014	0.004	0.001	0.025
Heterog	0.020	0.001	0.013	0.023	0.002	0.032	0.002	0.002
sfv	0.77	0.98	0.77	0.66	0.88	0.12	0.30	0.93

Spatial: spatially structured component variance.

Heterog: unstructured component variance.

Spatial fractional variance (sfv): var.RRspatial/(var.RRspatial + var.RRhet).

Additional file 1 includes the following information: 1) Probability density functions showing distribution of smoothed relative risks used in each map and 2) Plots showing the evolution of the variance of model spatial and heterogeneity components and the proportion of the mortality relative risk variance explained by the spatial structure of the models.

## Discussion

This study describes the mortality geographical patterns trend across four quinquennia, and so updates the municipal mortality maps previously published for the most frequent malignant tumours in Spain. Generally speaking, the mortality patterns observed for the most frequent cancers of the digestive system (stomach and colorectal) and respiratory tract, appear to be stable over time. In women, the distribution of mortality due to the tumours studied showed no defined spatial patterns, with the single exception of stomach cancer. However, time trends in spatial fractional variance enable the presence of changes in the spatial pattern to be assessed. Among the tumours analysed, breast, bladder and colorectal cancer in women suggest the possible appearance of a spatial pattern in the last quinquennium, a circumstance that will have to be monitored by subsequent studies. Prostate cancer mortality displayed no clear geographical pattern.

Cancer mortality is a dynamic process that is variable in time and space. Yet, the prolonged latency periods that exist between exposure and disease, mean that changes in incidence and/or mortality indicators may occur gradually over time, and their examination requires the incorporation of instruments of spatio-temporal analysis. In recent years, a number of spatio-temporal analysis techniques have been proposed [7–11]. The implementation of these methods is not simple, however, in that their theoretical development is far in advance of that of specific applications. Furthermore, there is no broad consensus as to how to describe spatial and temporal trends simultaneously and appropriately. These techniques’ performance might possibly be very different if they were applied to diseases without long latency periods.

In this study we opted to use separate spatial models for each period. Accordingly, each map shares the spatial data, and the temporal data are solely subject to the constraint of selection of reference rates for each period. This decision was taken after exploring other possibilities, such as plotting the maps by periods obtained on the basis of spatio-temporal models having a common reference rate or specific reference rates for each of the four periods. Each of these approaches has its advantages and disadvantages. The best solution is probably to adapt each method of presentation and modelling to the disease being studied, or to draw conclusions from the results of the different methods of analysis.

In Spain, cancer, both overall and by principal site (lung, colon-rectum, prostate, stomach, pancreas, breast, uterus, brain, leukaemia, lymphomas and myeloma), is well reflected in death certificates. A complete description of the entire process of certification and registration of deaths in Spain and an assessment of the quality of the information have been published elsewhere [18].

Since the development of a malignant tumour is a slow process, with a long latency period, influenced by many factors, the epidemiology of these tumours cannot be understood without taking into account the interaction between different environmental factors and genetic factors. On the other hand, spatial patterns could reveal differences in risk factors and make a significant contribution to establish policies for the fight against cancer in different areas and population groups.

### Stomach cancer

In Spain (2011), stomach cancer caused 5597 deaths, which accounted for 5.3% of all cancer-related deaths. It is estimated that 7810 new cases were diagnosed in 2012. In Spain, the adjusted incidence rate (European standard population) estimated for 2012 was 16.4 cases per 100,000 population in men (10.8 mortality) and 7.5 in women (4.8 mortality) [19]. In terms of mortality, the rates estimated for this same year were 10.8 per 100,000 population in men and 4.8 in women. The small difference between the incidence and mortality rates is due to the low survival recorded for gastric cancer, which is estimated in Spain to be 27.8% at 5 years, a little above the mean relative survival for Europe as a whole (24.1) [20]. Within Europe, the highest incidence is registered by Central and East European countries, while Nordic countries register the lowest rates for both sexes [21].

Until a few decades ago, gastric cancer was the most frequent cause of cancer-related death world-wide. Subsequently, albeit at different points in time, incidence and mortality due to this tumour began to decline in all countries during the second half of the 20^th^ century. Despite this decline, this tumour continues to rank fourth in incidence and the second in mortality [21]. Currently, the highest rates are registered in developing countries, and in East Asia in particular [21].

In Spain, there is a geographical gastric-cancer mortality distribution pattern that is characterised by its persistence in time and, in particular, by its similarity in both sexes. This study shows that the pattern has not changed substantially over the 20 years studied. Although the implication of *H. pylori* infection in the pattern observed in this country is uncertain, one cannot rule out the implication of different *H. pylori* strains which circulate in different regions or which circulated in the first half of the 20^th^ century when the generations currently studied were infected (during their infancy). Furthermore, the marked coast-interior pattern might be associated with different dietary habits in more rural areas of the interior, where more cured/smoked foodstuffs and less fruit and vegetables might be consumed than in coastal areas, or with territorially-related environmental exposures.

In order to understand the epidemiology of this tumour the interaction among the factors responsible for the virulence of the micro-organisms implicated, environmental factors and genetic factors [22] have to be taken into account.

The great geographical variability in this tumour’s mortality in Spain indicates the need to continue studying the factors implicated in the excess risk that is present in some areas, to enable its frequency to be reduced.

### Colorectal cancer

In Spain, colorectal cancer accounts for 13.7% of cancer-related deaths in men and 15.8% in women, according to data for the year 2011. Estimates for 2012 put the number of new cases in both sexes at 32,240, and the number of deaths at 14,700 [19]. The two sites are normally analysed jointly due to frequent errors of classification of tumours of the rectosigmoid junction [18]. Mortality is very high, with this constituting the second leading tumour site among men and women alike. From the second half of the 1990s onwards, mortality and incidence in both sexes were seen to level off [23].

In these tumours, the mortality data do not reflect the true incidence of the disease, since survival has improved in recent years, mainly among the young. In Spain, age-standardised relative survival at five years of diagnosis stands at 53.6% in both sexes, 54.9% if the tumour is located in the colon, and 51.7% if it is situated in the rectum [20].

In most cases, the aetiology of colorectal cancer is unknown. The frequency of these tumours has been linked to economic development, i.e., unlike the remaining tumours of the digestive system (buccal cavity and pharynx, oesophagus and stomach), it is highest in the most developed countries. Incidence and mortality rates in Spain are similar to the average rates for Europe and West European countries, with the highest rates corresponding to East European countries. In 2006, mortality and incidence were substantially lower in Spain than in North European countries, with Spanish rates being below the average for Europe.

Among the known aetiological factors is genetic predisposition, which would give rise to the presence of familial polyposis with tumours that more frequently turned malignant. Hereditary factors are present in 10% to 15% of cases. Risk factors described in the literature include higher consumption of meat and animal fats, and lower fibre consumption. In addition, a number of cohort studies, case-control studies and meta-analyses published in recent years have confirmed that smoking increases the risk of developing colorectal cancer [24, 25]. Although the results are not very consistent, alcohol consumption has been reported as being a possible risk factor [26]. Reported protective factors include intake of vegetables, fruit, fibre, calcium and some medications [27, 28]. The pathogenic mechanism postulated is the action of intestinal bacteria on biliary acids and fats producing carcinogenic substances. A diet poor in fibre would make for a slower gastrointestinal transit and thereby increase the degree of contact between such carcinogens and the mucosa [29]. Finally there are some studies suggesting that environmental factors like industrial emissions to air might be a risk factor for colorectal cancer [30, 31].

During the last five-year period studied, some Autonomous Regions (*Comunidades Autónomas*), acting in accordance with European Screening Guidelines [32], have implemented colorectal cancer screening programmes. This is the case of Catalonia, Valencian Region, Murcia, the Basque Country and Cantabria [33]. Although these programmes can be assumed to have had little or negligible influence on the results shown, they will contribute in the near future to reducing incidence and mortality due to this cause and could influence the spatial pattern of mortality if this practice is not extended to the whole population.

### Lung cancer

This is the most frequent tumour among men in Spain. In 2011, there were 17,485 male deaths due to this cause, accounting for 28% of all malignant tumours; in women there were 3,577 deaths due to this tumour, accounting for 5% of all cancers. Spain ranks midway in male incidence and mortality with respect to other countries in the region. Female incidence and mortality rates are still among the lowest in Europe, and Denmark has rates that are 4 times higher than those in Spain. Lung cancer prognosis is extremely poor, with relative age-adjusted survival at five years standing at 10.7% in Spain and generally lower in the other European countries [20].

Age-adjusted mortality rates among men peaked in 1995, with 72.5 cases per 100,000 population, and declined thereafter. Female adjusted rates began to rise at the end of the 1980s, and the trends in specific rates and the cohort effect are radically different to those in men, with a rise in risk being in evidence from the l940-generation onwards. This shows the effect of the smoking habit being taken up by women. The mortality trend is very different to that of other European countries and confirms the onset of the lung cancer epidemic among women in Spain.

Established environmental risk factors for lung cancer include: smoking cigarettes and other tobacco products; exposure to second-hand tobacco smoke; occupational exposure to benzo(a)pyrene and agents such as asbestos, nickel, chromium, and arsenic (inhalation and ingestion); exposure to radiation, including radon gas in homes and mines; and exposure to indoor and outdoor air pollution [34].

The spatial pattern of lung cancer mortality is assumed to be determined by the prevalence of smokers; any changes in this pattern over time would thus derive from the trend in this prevalence. Yet, visual examination of the spatial pattern shows it to be very similar across the four five-year periods for each sex separately, though a detailed study brings some variations to light. A certain attenuation of the RR would seem to be present among men in towns in west Andalusia. In women, the most notable finding was the excess risk observed for towns in Pontevedra and Ourense (Galicia) across all four quinquennia. This might be related to greater exposure to radon in homes in these areas, since maps of the local natural radiation sites coincide with those areas having the highest levels in Galicia [35].

Another finding of note was that in the last five-year period, excess risk among women centred on the most urban municipal areas. This excess risk points to provincial capitals and metropolitan areas where the prevalence of smokers is higher than that of rural areas, and where, moreover, the effect of air pollution would also have to be taken into account.

Recently, the International Agency for Research on Cancer (IARC) has concluded that there is sufficient evidence to show that exposure to outdoor air pollution causes lung cancer (Group 1). It has also noted a positive association with increased risk of bladder cancer [36]. Synergism between air pollution and smoking would indicate that the burden of cancer associated with smoking may be far greater than that indicated by the estimated risk attributable to smoking alone [36].

### Breast cancer

In Spain, some 26,000 breast cancer cases are diagnosed per year [37] and lead to the death of around 6000 women. The incidence rate estimated for 2012 was 85 cases per 100,000 women per year (standard European population), very similar to that of countries such as Portugal and Austria [19]. The adjusted mortality rate was 16.7 per 100,000, one of the lowest in Europe. It is estimated that in Spain there are currently more than 100,000 women with breast cancer who have been diagnosed in recent years [19].

Early detection programmes, together with diagnostic and therapeutic advances, have translated as an increase in survival, which exceeds 80% at five years of diagnosis [38]. Accordingly, mortality has lost validity when it comes to studying the frequency of the appearance of these tumours, though it continues to be the only indicator available for studying geographical variability across the nation. In Spain, breast cancer screening programmes were introduced in the various Autonomous Regions in the 1990s, and by 2001 had attained a coverage of over 90%. The high participation rates in these programmes, which in Spain are population-based, have resulted in a momentary fall in incidence at the point where they reach “saturation” [39].

The principal known risk factors for breast cancer are age, premature menarchy, late-onset menopause, nulliparity or first pregnancy at late age, exposure to ionizing radiations, presence of mutations in high-penetrance genes (BRCA1, BRCA2 and others), family history [40], alcohol consumption and obesity in postmenopausal women [41]. Apart from factors linked to genetic susceptibility, the effect of the other risk determinants is explained in terms of cumulative lifetime oestrogen exposure [42]. Exogenous oestrogens also modify risk, with hormone replacement therapy being especially relevant, bearing in mind the point in time at which exposure occurs [43]. However, this type of treatment has been used very little in Spain [44]. Breast cancer describes a positive socio-economic gradient, with incidence and mortality being higher in groups having a higher socio-economic level [45]. This gradient is less evident in recent years, since it is due, in part, to the distribution of risk factors of a hormonal or reproductive nature in the different social levels.

### Prostate cancer

In 2012, Spain, with an estimated rate of 96.8 new cases per 100,000 population, had intermediate incidence rates with respect to Europe, while its mortality rates were the lowest on the continent. Even so, prostate cancer caused close on 1 of every 10 male cancer-related deaths in this country, ranking third, after lung cancer and colorectal tumours, as a tumour-related cause of death in Spanish men. The 2011 mortality rate in Spain was 17.11 deaths per 100,000 population. The mortality rates are considerably lower than the incidence rates, in view of this tumour’s good survival rate, which in Spain is around 75.4% at 5 years [20].

Prostate cancer incidence has increased in recent years [46], probably due to non-systematic PSA screening for this tumour. Mortality rates rose until 1995, something that is attributed to improvements in diagnosis and certification of cause of death. In recent years, the trend has become inverted, due to improvements in the survival of these patients. Although the spatial pattern in this tumour is not particularly noteworthy, the maps make it possible to discern a growing uniformity across the country, which means that in the last period there were no observable differences in risk between some regions and others. Indeed, the spatial fractional variance figures are the lowest of all the tumours studied in this analysis.

Neither the agents which cause the appearance of prostate cancer nor those which cause tumours to progress towards a clinically manifest tumour are known. Several polymorphisms associated with a higher risk of developing this cancer have already been identified [47]. Epidemiological, clinical and basic research has suggested that androgens might play an important role in prostatic carcinogenesis [48]. Glucose metabolism might be implicated in this tumour, which would explain why diabetics have a lower risk [49], and drugs such as statins might also possibly reduce the probability of developing an aggressive tumour [50]. With regard to diet, available information suggests that dairy products could increase prostate cancer risk [51], whereas foods containing lycopene or selenium might reduce it [41].

Insofar as the spatial pattern is concerned, none of the risk factors discussed (genetic susceptibility, hormones, diet or consumption of dairy products) is territorially related. Diabetes or the protective factor afforded by oral antidiabetics may perhaps account for the lower prostate cancer mortality observed for Andalusia, since in the period 1989-1998 this region had a higher diabetes-related mortality rate than did the rest of Spain [3].

### Bladder cancer

In a European context, Spain regrettably occupies a pre-eminent position in terms of this tumour’s incidence (1^st^ country) and mortality (2^nd^ country): in 2011 there were 4,153 bladder cancer-related deaths in men and 931 in women, with the male and female European-population-adjusted mortality rates being 12.81 and 1.95 per 100,000 population respectively. It is estimated that in 2012 there were 13,789 new cases en Spain, with partial prevalence (cases diagnosed in the last 5 years) being very high, i.e., 47,225 cases, ranking 4^th^ after breast, prostate and colorectal cancer. Relative survival for this tumour in Spain at 5 years of diagnosis is 74.09% in men and 72.50% in women, somewhat higher than that for Europe as a whole [20]. This frequency, coupled with the relapsing nature of bladder cancer, means that it place an enormous burden on health care systems [52, 53].

Smoking is recognised as the most important risk factor for bladder cancer and is estimated to account for 50% of tumours. Tobacco smoke contains aromatic amines, such as b-naphthylamine, and polycyclic aromatic hydrocarbons known to cause bladder cancer [54]. These are renally excreted and exert a carcinogenic effect on the entire urinary system. After smoking, occupational exposure to carcinogens -namely, aromatic amines, polycyclic aromatic hydrocarbons, and chlorinated hydrocarbons- is viewed as the second most important risk factor. It has been suggested that roughly 20% of all bladder cancers are related to such exposure, mainly in industrial areas producing and processing paint, dye, metal, and petroleum products. As for other cancers, nutritional aspects have been attributed to bladder cancer risk [52]. Exposure to arsenic in drinking water has been recognised as a cause of bladder cancer. In general, the differences in incidence and mortality by gender, race or socio-economic status reported in other studies can be explained by differences in habits or by other exposures.

Increasing evidence suggests that genetic predisposition has a significant influence on incidence, especially through its impact on susceptibility to other risk factors. The frequency of bladder cancer will nevertheless remain relatively unaffected due to the ongoing high prevalence of smoking, its main risk factor [52].

The pattern of bladder cancer in men displays some analogies with that of lung cancer, though it also reveals notable differences. The similarities are the higher risk in many towns situated in the provinces of Cadiz, Huelva and Seville, in Almería, and along the coast of the Valencian Region. The most marked contrasts with the pattern plotted by lung cancer are to be found in Extremadura, Asturias and the province of Barcelona. Towns in Cadiz, Seville and Huelva display excess risk in both sexes. The coincidence with excess risk of lung cancer among men across large parts of the country, points to the effect of smoking. In view of the singular spatial distribution of these towns, it would seem that determinants other than the high prevalence of smokers are present in the pattern of bladder cancer mortality in this area, since this same pattern is also observed in women in the first five-year period. There have been reports of excess risk in districts of Catalonia and its possible relationship with mining activity and the textile industry. A fuller discussion of these results, which includes suggestions of different hypotheses that seek to explain these patterns, may be consulted in a previous article [14]. The maps show a change in the distribution of mortality in women, characterised by the appearance of excess mortality in the last five-year period in towns in Catalonia, Aragon and Navarre, as well as in Valencia and Alicante, which is probably linked to the increase in the prevalence of female smokers in these areas.

## Conclusions

Among men in Spain, geographical patterns of mortality due to the most frequent cancers of the digestive system (stomach and colorectal), lung cancer and bladder cancer seem to be stable over time. Prostate cancer and the tumours studied in women show no defined spatial pattern, with the single exception of stomach cancer. Breast, colorectal and bladder cancer in women show signs of the possible appearance of a spatial pattern in the last five-year period, and should therefore be monitored.

## Electronic supplementary material

Additional file 1: **Additional file 1 includes the following information: 1) Probability density functions showing distribution of smoothed relative risks used in each map and 2) Plots showing the evolution of the variance of model spatial and heterogeneity components and the proportion of the mortality relative risk variance explained by the spatial structure of the models.** (PDF 184 KB)
