# Supplementary material for: Time trends in municipal distribution patterns of cancer mortality in Spain
Source: BMC Cancer. 2014 Jul 24;14:535. doi: 10.1186/1471-2407-14-535 (PMC4124154; doi:10.1186/1471-2407-14-535)

Supplementary material of the article: **Time trends in municipal distribution patterns of cancer mortality in Spain.**

Supplementary material includes the following information: 1) Probability density functions showing distribution of smoothed relative risks used in each map and 2) Plots showing the evolution of the variance of model spatial and heterogeneity components and the proportion of the mortality relative risk variance explained by the spatial structure of the models.

Figure-s1. Probability density functions showing distribution of smoothed relative risks used in each map

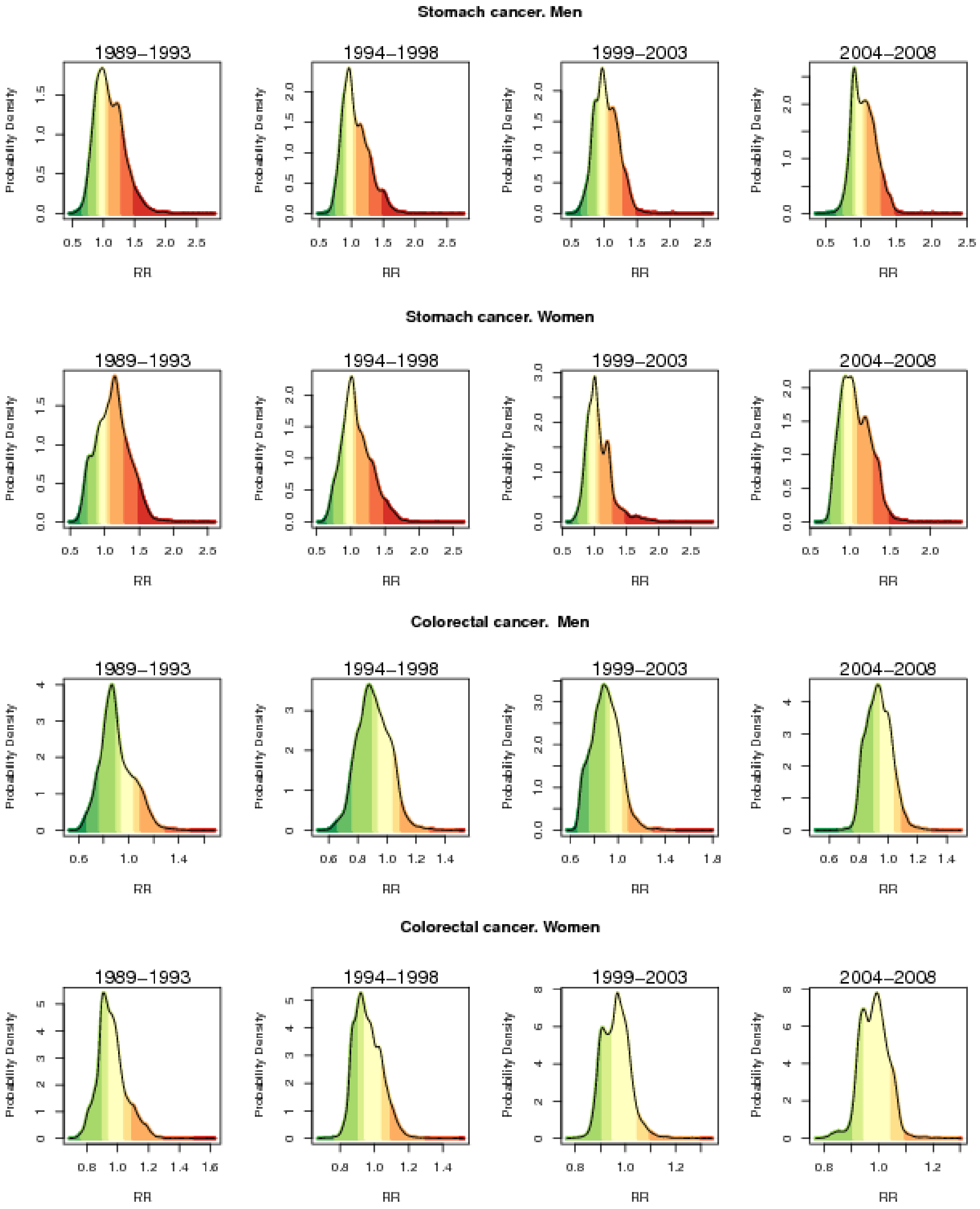

Lung cancer. Men

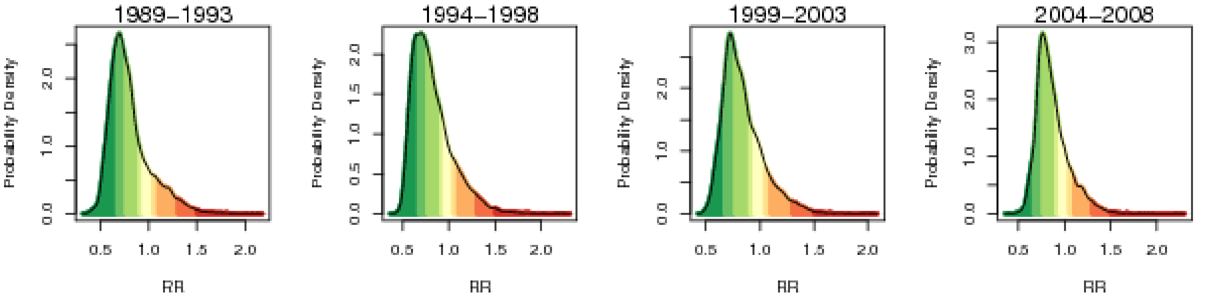

Lung cancer. Women

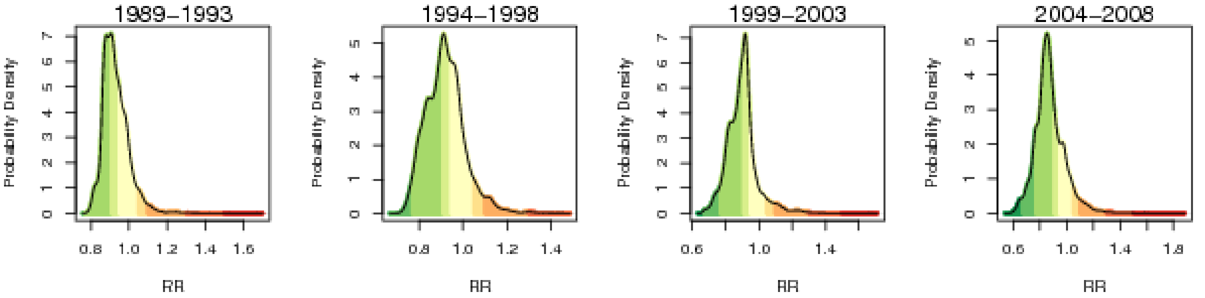

Breast cancer. Women

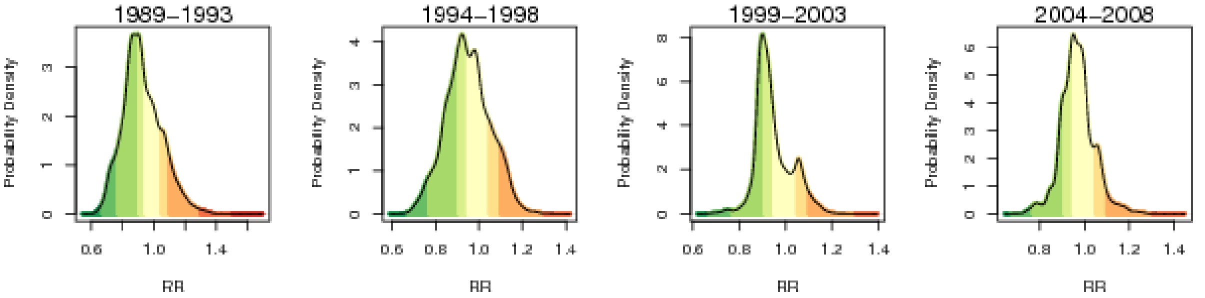

Prostate cancer

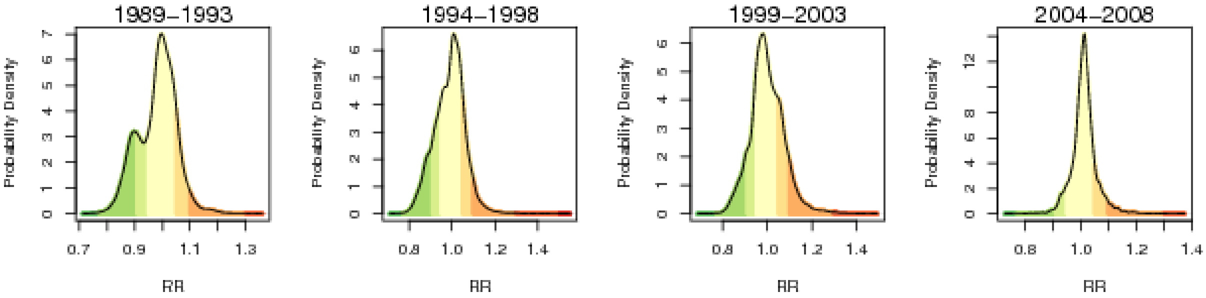

Bladder cancer. Men

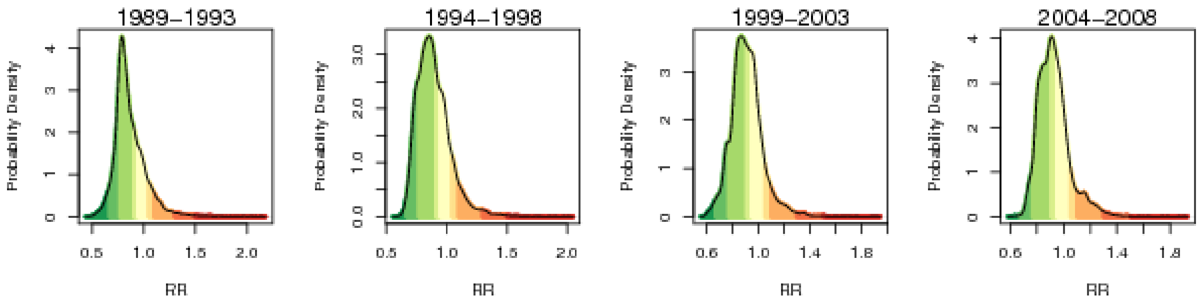

Bladder cancer. Women

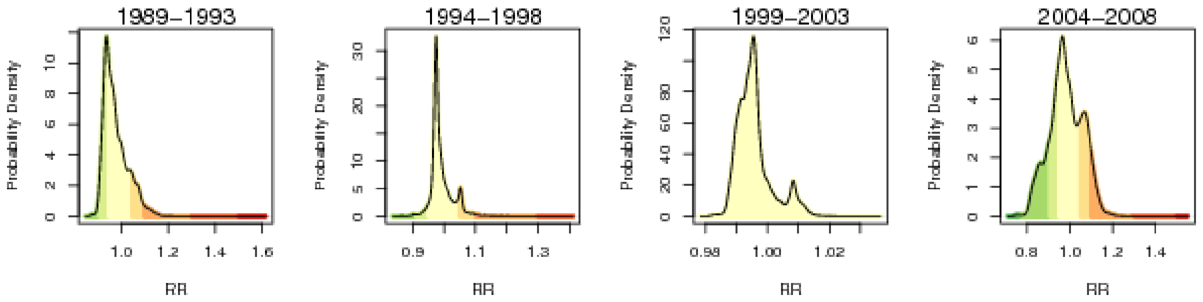

Figura-s2. Plots showing the evolution of the variance of model spatial and heterogeneity components and the proportion of the mortality relative risk variance explained by the spatial structure of the models.

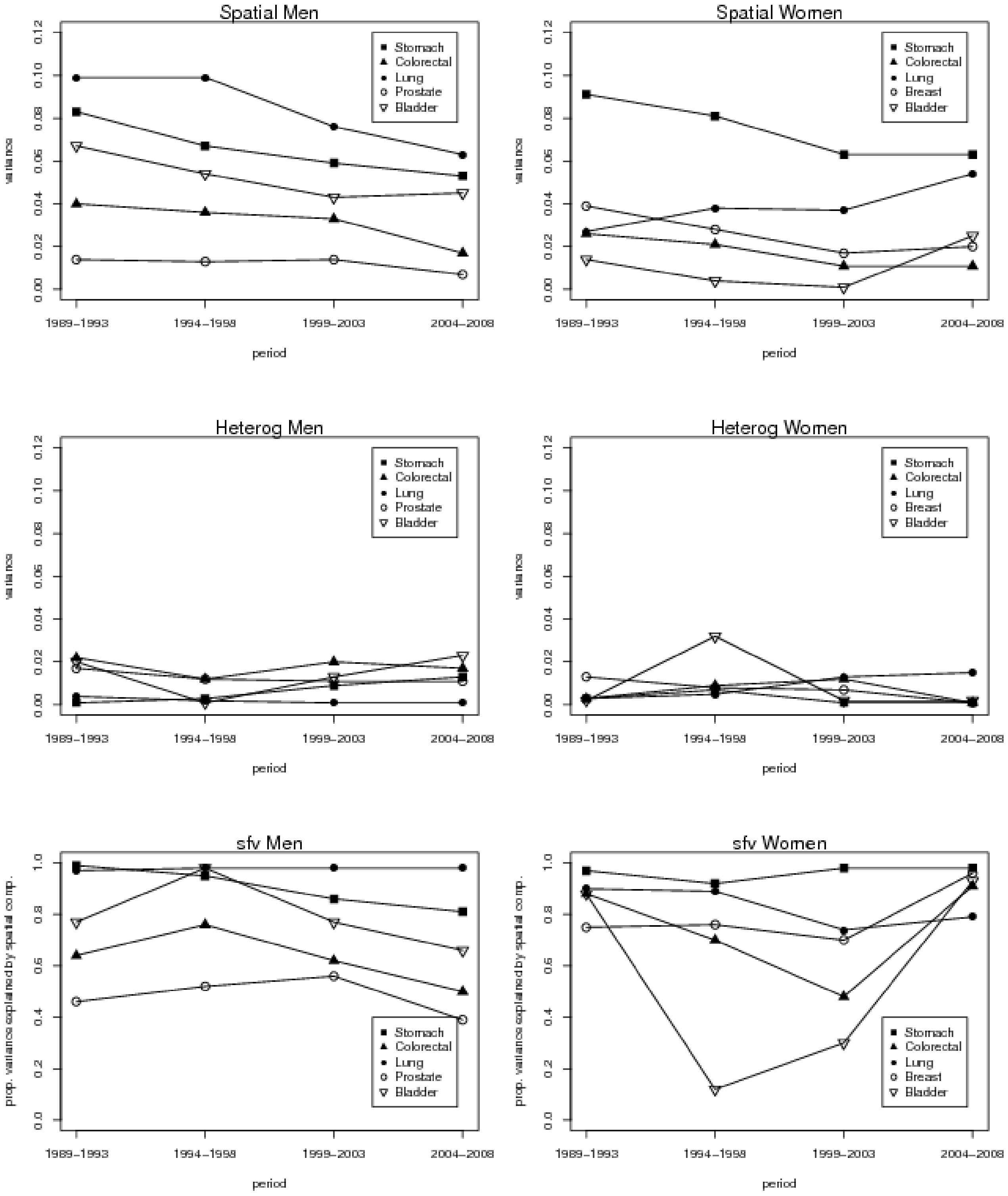

Supplement: Supplementary file 1 — Additional file 1: Additional file 1 includes the following information: 1) Probability density functions showing distribution of smoothed relative risks used in each map and 2) Plots showing the evolution of the variance of model spatial and heterogeneity components and the proportion of the mortality relative risk variance explained by the spatial structure of the models. (PDF 184 KB) [file 12885_2014_4728_MOESM1_ESM.pdf]
